# Metagenome-Assembled Genome Sequences Recovered from Epilithic River Biofilm in the Tama River, Japan

**DOI:** 10.1128/MRA.00664-21

**Published:** 2021-09-23

**Authors:** Joval N. Martinez, Arisa Nishihara, Shin Haruta

**Affiliations:** a Department of Biological Sciences, Tokyo Metropolitan University, Hachioji, Tokyo, Japan; b Department of Natural Sciences, University of St. La Salle, Bacolod City, Negros Occidental, Philippines; c Bioproduction Research Institute, National Institute of Advanced Industrial Science and Technology (AIST), Tsukuba, Ibaraki, Japan; Montana State University

## Abstract

Draft genome sequences of putatively novel bacteria were assembled from the metagenome of epilithic biofilm samples collected from the Tama River (Tokyo, Japan). The metagenome contains 44,630,724 sequences, 44,792 contigs, and 48% G+C content. Binning resulted in 31 metagenome-assembled genomes (MAGs) with ≥50% completeness.

## ANNOUNCEMENT

Epilithic biofilm sustains river ecosystems by producing organic substances and degrading organic matter. The main components of the riverbed biofilm in clear streams are microscopically identified as oxygenic photosynthetic organisms (1), but their metabolic potentials and other diverse coexisting microbes have not been studied well. Hirose et al. reported a PCR amplicon analysis of riverbed biofilm from the Tama River, a major river flowing through the Tokyo area, Japan, and found an unexpected diversity of anoxygenic phototrophic bacteria (2, 3). Here, we report the metagenome-assembled genome sequences (MAGs) retrieved from metagenomic reads of the biofilm in the Tama River.

Epilithic biofilms that had developed on a stone in the riverbed were collected from the Tama River, Ome City, Tokyo, Japan (35°47′10.6″N, 139°15′16.5″E), on 23 November 2014. Biofilms were scraped off the stone, placed into one 1.5-ml collection tube using a sterilized cotton swab, stored on ice during transportation to the laboratory (1 h), and stored at −20°C until further use. DNA from the biofilm sample was extracted and purified using the PowerBiofilm DNA isolation kit (Qiagen) for metagenomic sequencing. Sequencing libraries were prepared using the Illumina TruSeq library prep kit. A total of 90,614,554 bp metagenome reads from paired-end sequencing (2 × 101 cycles) were quality filtered using the Illumina chastity filter (Hokkaido System Science Co., Ltd.) and assessed using FastQC v.1.1.1 (4). Metagenome analyses were implemented using DOE Systems Biology Knowledgebase (KBase) (5). A total of 44,630,724 sequences were retrieved from the trimming process using Trimmomatic v.0.32 (6). These were assembled as contigs using metaSPAdes v.3.13.0 (7). Quality assessment using QUAST v.4.4 (8, 9) resulted in a total of 44,792 contigs with 48% G+C content. The contigs were binned and optimized using Concoct v.1.3.4 (10). Optimization of binning resulted in 71 putative draft metagenome-assembled genome sequences (MAGs), in which 31 bacterial MAGs were ≥50% complete with mostly ≤3% contamination as determined using CheckM (11). Default parameters were employed for all the software used.

Based on taxonomic assignments using the Genome Taxonomy Database Toolkit (GTDB-Tk) Classify v.0.1.4 (12), 31 river biofilm (RB) MAGs were classified as members of the phyla *Proteobacteria* (RB00, RB05, RB08, RB09, RB15, RB24, RB29, RB30, RB37, RB44, RB52, RB72, RB77, RB81, RB82, RB83, RB85, RB89, RB90), *Bacteroidota* (RB06, RB13, RB22, RB46, RB50, RB56, RB76, RB88), *Verrucomicrobiota* (RB28, RB53, RB61), and *Cyanobacteria* (RB71). All MAGs recovered had ≤97% average nucleotide identity (ANI) compared to the closest cultured relatives, suggesting that the bacteria which harbored these MAGs are potentially novel species (13). The MAGs were annotated using Prokka v.1.14.5 (14). The annotated putative draft genome sequences contain *pufLMC* genes encoding photosynthetic reaction centers, assigned to only two MAGs belonging to the classes *Alphaproteobacteria* (RB90) and *Gammaproteobacteria* (RB00). Genes encoding proteorhodopsin were also recovered from four MAGs representing *Bacteroidota/Chlorobi* (RB24, RB50, RB56, RB88), supporting findings for the widespread distribution of these light-driven proton pumps in freshwater ecosystems (15). As for the nitrogen metabolisms, no homologous gene involved in nitrogen fixation (*nifHDKEN*) was recovered in the metagenome. However, two denitrification-related genes, *narG* and *nosZ*, were retrieved from MAGs belonging to the classes *Gammaproteobacteria* (RB82) and *Bacteroidia* (RB56). Genes encoding reactive oxygen species (ROS) (e.g., superoxide dismutase, peroxidases) were recovered from 12 MAGs, aiding in protection and survival against oxidative stress, similar to other heterotrophic bacteria (16).

### Data availability.

This whole-genome shotgun project has been deposited at DDBJ/ENA/GenBank under the BioProject accession number PRJNA668882. The raw sequence reads are available in the Sequence Read Archive (SRA) under the accession numbers SRR13089527 to SRR13089538. The MAGs are accessible at https://kbase.us/n/95585/4/.
